# Durability of Construction and Demolition Waste-Bearing Ternary Eco-Cements

**DOI:** 10.3390/ma15082921

**Published:** 2022-04-16

**Authors:** Jaime Moreno-Juez, Laura Caneda-Martínez, Raquel Vigil de la Villa, Iñigo Vegas, Moisés Frías

**Affiliations:** 1 Tecnalia, Basque Research and Technology Alliance (BRTA), Astondo Bidea, Edificio 700, Parque Tecnológico de Bizkaia, 48160 Derio, Spain; jaime.moreno@tecnalia.com (J.M.-J.); inigo.vegas@tecnalia.com (I.V.); 2 Eduardo Torroja Institute for Construction Science (IETcc-CSIC), 28033 Madrid, Spain; laura.cmartinez@udc.es; 3 Departamento de Geología y Geoquímica, Geomateriales Unidad Asociada CSIC-UAM, Universidad Autónoma de Madrid, 28049 Madrid, Spain; raquel.vigil@uam.es

**Keywords:** construction and demolition waste, concrete fines, glass, binary pozzolanic blend, ternary cement mortars, external agents, durability

## Abstract

In recent years, the development of ternary cements has become a priority research line for obtaining cements with a lower carbon footprint, with the goal to contribute to achieve climate neutrality by 2050. This study compared ordinary Portland cement (OPC) durability to the performance of ternary cements bearing OPC plus 7% of a 2:1 binary blend of either calcareous (Hc) or siliceous (Hs) concrete waste fines and shatterproof glass. Durability was measured further to the existing legislation for testing concrete water absorption, effective porosity, pressurized water absorption and resistance to chlorides and CO_2_. The experimental findings showed that the 7% blended mortars performed better than the reference cement in terms of total and effective porosity, but they absorbed more pressurized water. They also exhibited lower CO_2_ resistance, particularly in the calcareous blend, likely due to its higher porosity. Including the binary blend of CDW enhanced chloride resistance with diffusion coefficients of 2.9 × 10^−11^ m^2^ s^−1^ (calcareous fines-glass, 7%Hc-G) and 1.5 × 10^−11^ m^2^ s^−1^ (siliceous fines-glass, 7%Hs-G) compared to the reference cement’s 4.3 × 10^−11^ m^2^ s^−1^. The siliceous fines-glass blend out-performed the calcareous blend in all the durability tests. As the mortars with and without CDW (construction and demolition waste) performed to similar standards overall, the former were deemed viable for the manufacture of future eco-efficient cements.

## 1. Introduction

Given the vast volumes of construction and demolition waste (CDW) generated yearly, it is one of the streams of refuse presently attracting the interest of both the scientific community and the construction industry. The 900 million tonnes of CDW produced in Europe in 2015 [1] accounted for one-third of all the waste generated in the region that year. Although a mean 35% was valorized in a number of industrial applications [2], the rest posed, and continues to pose, severe technical, economic, environmental and social problems [3]. In response to those issues, solutions have been proposed to improve construction industry sustainability [4] in keeping with circular economy strategies, such as Agenda 2030 and others, geared to attaining climate neutrality by 2050. The ultimate goal is to transition from today’s linear economy and the use of natural resources to circular arrangements with the reuse of industrial waste as secondary raw materials. 

After processing in management plants, CDW is divided into essentially two types of inorganic waste: concrete and mixed (concrete + masonry), whose deployment has been explored for primarily three applications. These include (a) coarse recycled aggregate for concrete [5,6,7], a use envisaged in many national standards, including Spain’s structural concrete code [8], for concrete waste in particular at replacement ratios of up to 20%; (b) recycled fines in mortars and concretes [9,10,11]; and (c) supplementary cementitious materials (SCMs) for the manufacture of future eco-cements with a smaller carbon footprint and lower Portland clinker content [12,13,14,15].

Under the third area, recent research has focused on recycled concrete fines (<5 mm), a material generated during concrete waste crushing and milling. That by-product is subsequently stockpiled in management plants, given its non-reusability due to the possible presence of very fine particles or even deleterious (sulfate, chloride) materials. Preliminary research conducted by the multidisciplinary team of authors of this article showed that these fine concrete fractions have low-to-medium pozzolanicity depending on factors such as the calcareous or siliceous nature of the original aggregate, the formation of C-S-H, C_4_AH_13_ and C_4_AcH_12_ gels [16] and the optimal physical and mechanical performance (or otherwise) of the 7% blended cement used [17,18,19]. Subsequent studies with binary blends prepared from the same concrete fines plus shatterproof glass (likewise recycled from CDW) at ratios of 2:1, 1:1 and 1:2 by weight revealed greater pozzolanicity (reactivity with lime in the medium) than exhibited by the same waste without glass [17]. Reaction kinetics, in turn, were found to depend on the nature of the concrete waste [20]. In all cases, the ternary cement pastes were observed to form more polymerized C-S-H gels than OPC with different morphologies and sodium in their structure due to the release of that ion by the glass (Na_2_O = 13%). Such microstructural changes would necessarily have a direct impact on the performance of these new ternary eco-cements.

The present study therefore proceeded to a first-ever analysis of the durability of ternary cement mortars made with 7% (2:1) concrete waste/glass blends based on their performance in terms of total water absorption, effective porosity, pressurized water penetration and carbonation and chloride resistance. 

## 2. Materials and Methods

### 2.1. Raw Materials

The OPC used in this study, classified in Spanish legislation as CEM I 52.5R [21], was supplied by Cementos Lemona, S.A. (Bilbao, Spain). 

**Figure 1:**
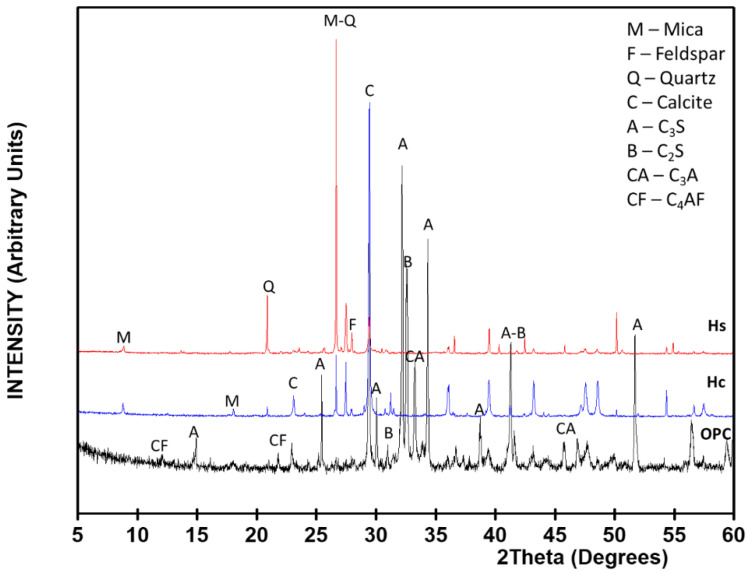
XRD patterns of the materials analyzed.

Three construction and demolition wastes were analyzed: Two wastes from two fractions of fines (<5 mm) resulting from concrete waste crushing and milling were taken from two Spanish waste management plants that had stockpiled the material in open yards. Depending on the nature of the original aggregate, such fines were divided into calcareous (Hc) and siliceous (Hs). Finally, a shatterproof glass (G) with 100% amorphous material taken from a demolished residential building in northern Spain was selected. All three types of CDW were ground in the laboratory to a particle size of under 63 µm. X-ray diffraction (XRD) patterns and quantitative Rietveld analysis of the raw materials analyzed are included in Figure 1 and Table 1.

### 2.2. Ternary Cement Preparation

**Figure 2:**
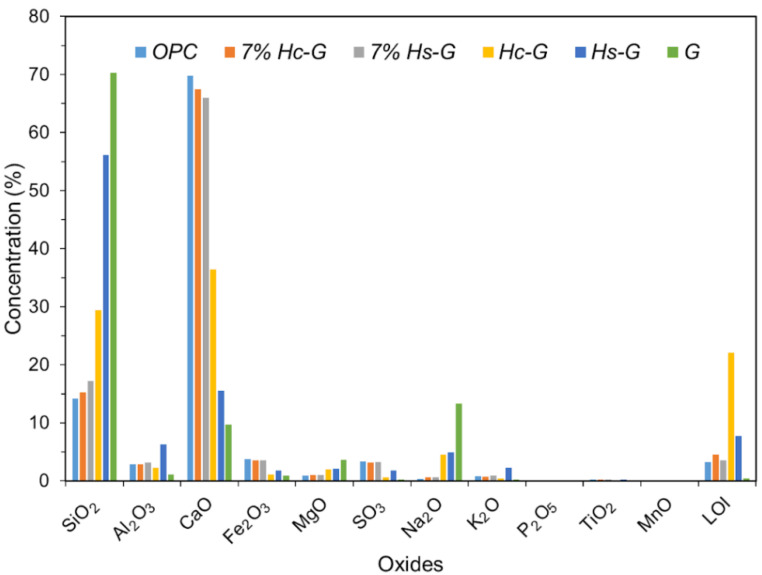
XRF-determined chemical composition of the samples analyzed.

The ternary eco-cements were prepared by replacing 7% of the OPC with a 2:1 (by weight) binary blend of either Hc-G or Hs-G, found in preliminary research to be the optimal proportion [18,20]. The cements studied were labelled OPC (reference cement), 7% Hc-G (OPC additioned with 7% 2:1 Hc-G) and 7% Hs-G (OPC with 7% 2:1 Hs-G). XRF-determined chemical composition of the cements analyzed are included in Figure 2, whilst Table 2 gives their D10, D50 and D90 values.

The blended cement mortars were prepared with standardized siliceous sand (≥98% SiO_2_) at a binder/sand ratio of 1:3 and a water/binder ratio of 0.5 as specified in European standard EN 196-1 [22]. Prior to analyzing their physical properties, all the mortar specimens were soaked in water for up to 28 d. They were subsequently dried and tested further to the standards listed in the following subsection.

### 2.3. Methods

The durability tests were conducted to the recommendations set out in the existing standards for hardened concretes.

Water absorption, density and effective porosity, were found in keeping with Spanish standard UNE 83980 [23] on two samples drawn from the same specimen, core or test piece. Accordingly, the samples were oven dried to a constant weight and then immersed in water for 48 h. After determination of their weight, the specimens were boiled for 5 h. Subsequently, the specimens were allowed to cool to room temperature and were weighed in air and in water on a hydrostatic scale.

Pressurized water penetration was conducted as described in Spanish and European standard UNE EN 12390-8 [24] by hosing a 150 mm cubic specimen with water at a pressure of 500 kPa for 72 h. The specimens were split in two halves and the maximum and average depth of water penetration was then measured.

To find carbonation resistance as specified in international standard ISO 1920-12 [25], specimens were conditioned in a laboratory air environment for 14 days, and then exposed to accelerated carbonation with 3% CO_2_ for 72 d at 65% R.H. Carbonation depth, in turn, was determined by exposing two 100 mm cubic specimens to the phenolphthalein test.

Chloride ion resistance was established further to Spanish/European standard UE EN 12390-11 [26] on unidirectional diffusion. Accordingly, two 100 × 100 mm^2^ cylindrical specimens were water-saturated and provided with a pond containing 3% NaCl solution on one of their circular surfaces, sealed with a plastic film. The specimens were kept in these conditions for 90 days. After the exposure period, powder samples were extracted at different depths and their chloride content was subsequently analyzed by titration with silver nitrate according to the Volhard method (Method A), as described in the Spanish/European standard UNE EN 14629 [27].

### 2.4. Characterization Techniques

The compressive strength of the mortars was determined on an IBERTEST AUTOTEST 200/20-SW press on 28-day mortar specimens, following the EN 196-1 standard [22].

The chemical composition of the main oxides present was determined on pressed powder wafers using a Philips PW-1404 (Philips, Madrid, Spain) X-ray fluorescence spectrometer fitted with an Sc-Mo X-ray tube. 

Mineralogical analysis and crystalline phase quantification were conducted on a Malvern Panalytical X’Pert Pro (Madrid, Spain) X-ray diffractometer bearing a copper anode and a 9.5 mm receiving slit, operating at 40 mA and 45 kV. The measuring range was 5–60°, with rutile as the internal standard. Rietveld quantification was calculated with Match (v.3) and Fullprof software (Bonn, Germany).

The dry dispersion D10, D50 and D90 values for the cements were found with a Malvern Panalytical Mastersizer 3000 laser diffraction particle size analyzer.

Cement mortar morphology, analyzed after the accelerated carbonation test, was determined under an FE Inspect W-SEM/EDX electron microscope fitted with a DX4i analyzer and a Si/Li detector (Hillsboro, OR, USA).

## 3. Results and Discussion

### 3.1. Water Absorption, Density, Effective Porosity and Mechanical Strength

Table 3 Total post-soaking absorption (TSA), post-soaking and boiling absorption (TSBA), dry (DD) and bulk (BD) density and effective porosity (EP) found as specified in Spanish standard UNE 83980 [23].

The dry and bulk density values for the ternary cement and reference mortars were very similar, although slightly higher in the 7% additioned materials, in all likelihood as a result of their greater fineness, which would induce minor matrix densification (Table 2). 

Total post-soaking (TSA) and post-soaking and boiling (TSBA) water absorption values were lower in the mortars bearing 7% CDW-based binary blends than in the reference cement material. At 17%, the reduction in water absorption relative to the reference was greater in siliceous than in calcareous concrete waste, where the decline recorded was 9.2%.

Both blended cement mortars performed well in terms of post-soaking water absorption, with values much lower than the recommended ceiling (<10%) for high-quality cement matrices [28]. The explanation lies in the binary blend of pozzolans comprising low reactivity concrete waste (Hc: 1.18 × 10^−4^ K·h^−4^; Hs: 6.6 × 10^−4^ K·h^−4^) and high reactivity glass waste (G: 1.14 × 10^−2^ K·h^−1^). The resulting synergies were more intense in the siliceous (Hs) than in the calcareous concrete (Hc) blend. The pozzolanic reaction is widely known to induce additional hydrated phases in cement matrices, substantially modifying microporosity and the paste-aggregate interfacial transition zone (ITZ) [29,30]. The present findings were consistent with results reported in that connection by other authors working with blended cement mortars prepared with thermally activated coal tailings [31].

The standardized effective porosity (EP) test findings followed the same pattern as for TSA and TSBA, i.e., with the inclusion of the binary blend of pozzolans associated with declines in porosity of 5.2% in 7% Hc-G and 8.7% in 7% Hs-G relative to the reference mortar. As with total absorption, higher blended mortar performance in effective porosity was attendant upon the formation of secondary hydrated phases during the pozzolanic reaction. Vigil et al. [20] reported that the most prominent phase generated in pure Hs-G and Hc-G/lime systems, C-S-H gels, precipitated inside pores. The resulting reduction in microporosity and interconnectivity induced denser, less porous and less water-accessible mortar matrices.

In terms of mechanical properties, the 28-day compressive strength values shown in Table 3 reveal that the use of the binary CDW mixes at 7% substitution has a slightly negative effect. Nevertheless, only a minor impact on the mechanical performance is detected, reaching a loss of approximately 4% compared to the OPC mortar for both siliceous and calcareous mixes.

### 3.2. Water Penetration under Pressure

The penetration depths for the three mortars analyzed are summarized in Table 4. Greater depths were recorded for the mortars bearing 7% of either of the binary blends of pozzolans than for the reference mortar. The maximum value was 47.4% deeper in the former, irrespective of the nature of the original aggregate (Hc or Hs), with an absolute value of 28 mm in both types of blended mortar.

Further to the provisions of Spain’s Royal Decree on bulk and reinforced concrete (Spanish initials, DEE) [32], the mortars analyzed would be deemed impermeable to water under pressure, for the values observed lay below the allowable ceiling.

### 3.3. Accelerated Carbonation Resistance

**Figure 3:**
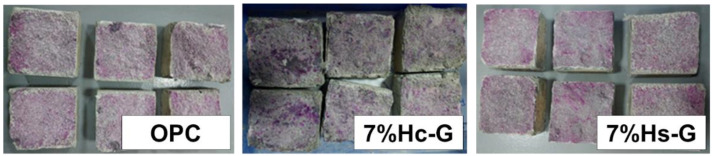
Carbonation front after 72 d exposure to 3% CO_2_.

Photographs of the mortar specimens after the accelerated carbonation test (72 d of exposure to 3% CO_2_) and spraying with phenolphthalein are reproduced in Figure 3.

Two clearly distinguishable areas were detected: the reddish-purple zone denoted pH values of over 10, an indication that the cement matrix was unaffected by the CO_2_, and a colorless rim denoting pH values of <8, signifying carbonation (primarily of portlandite) further to the following reaction:

CO_2_ + Ca(OH)_2_ → CaCO_3_ + H_2_O (1)

**Figure 4:**
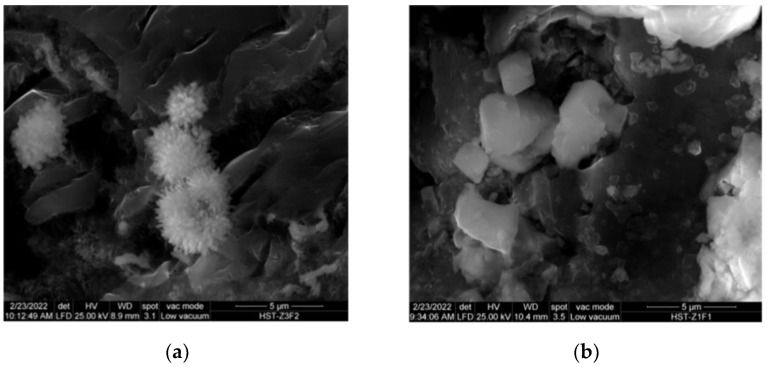
Carbonate morphology: (**a**) aragonite; (**b**) calcite.

However, other calcareous hydrates as well as anhydrous phases may have also been impacted by carbonation [33]. Portlandite carbonation is essentially a three-stage process: (a) portlandite dissolution; (b) absorption of CO_2_ and carbonate ion formation; and (c) chemical reaction and carbonate precipitation [34]. The SEM micrographs of the carbonated areas in the three mortars confirmed such carbonate precipitation in the form of aragonite and calcite (Figure 4).

The mean of the carbonation front depths measured on the two sides of each of the three specimens tested are given in Table 5, along with the respective standard deviations (SD).

After a 72-d exposure to 3% accelerated carbonation the mortars prepared with a binary blend of pozzolans exhibited lower carbonation resistance than the reference material, with 37.5% (7% Hc-G) and 15% (7% Hs-G) deeper carbonation fronts. The calcareous blend, in turn (Hc-G), was nearly 20% less resistant than the siliceous material (Hs-G). That may have primarily been the result of the higher effective porosity in the former (Table 3), which would have favored the uptake of the aggressive agent. It might also have been induced by the fact that the Hc waste was calcite-high (52%), which in turn would be associated with enhanced CO_2_ and calcium carbonate solubility due to the rise in the alkali content in the cement matrix prompted by the presence of glass [34].

Such poorer chemical resistance to carbonation in the blended cements would be associated with their lower portlandite content attributable to dilution (7% less clinker) and the pozzolanic reaction characteristic of CDW [16,35]. Both factors lead to a reduction of the alkaline reserve, which facilitates the lowering of the pH by reaction with CO_2_ and thus the progression of the carbonation front to deeper levels. In this study, a third factor may have had a further adverse impact on carbonation rate, attributable to the presence in the bended cements of recycled glass, known to have a high sodium content (recycled glass = 13% Na_2_O). Some authors have observed that alkalis favor carbonation [11], an adverse effect likewise reported for other pozzolanic additions (silica fume, fly ash, slag, activated paper, calcined clay, sludge, mixed CDW) [11,36,37,38,39].

### 3.4. Resistance to Chloride Ion Penetration

**Figure 5:**
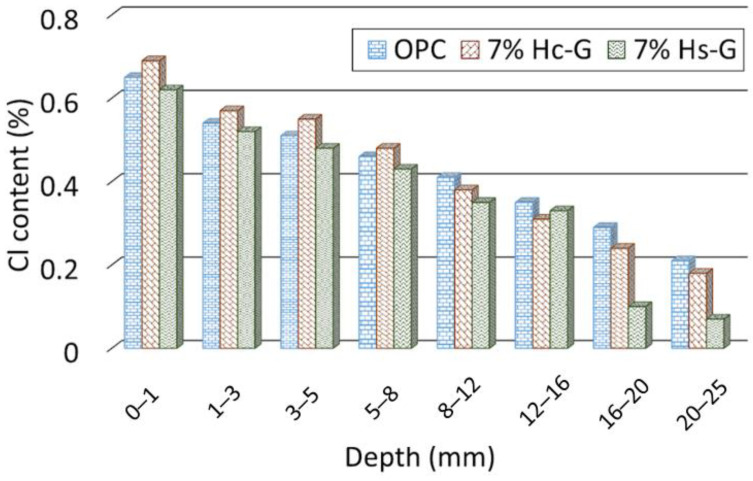
Variation in chloride ion concentration with depth.

Figure 5 graphs the variation in chloride ion content with depth in the three mortars studied. The baseline chloride content, essentially sourced from the Portland cement, was very similar in all three (0.02 wt % to 0.03 wt %). Although the decline in chloride ion concentration observed is characteristic of ion transport in cement-based materials, the three mortars behaved differently. At the deeper depths analyzed, both 7% Hs-G and 7% Hc-G had lower chloride concentrations than OPC, an indication of higher resistance to ion transport. The difference was particularly visible in 7% Hs-G, where concentration in the deepest layer was 67% lower than observed in the OPC mortar, whereas the decline in concentration recorded in 7% Hc-G mortar was less significant in that layer. The chloride ion concentration in the reference was higher than in the 7% Hs-G mortars in all the layers analyzed, but not in the 7% Hc-G mortars, where it was lower in the outermost (first 8 mm) but higher in the deeper layers (>8 mm). The inference is that the mechanism leading to a lower chloride ion transport rate varied with the type of pozzolan. 

The effect of the pozzolans on chloride ion transport was quantified by calculating the non-steady state diffusion coefficient (Dnss) for each mortar type. The experimental curves for chloride were subsequently fitted to Equation (2) in keeping with Fick’s law of diffusion as specified in Spanish/European standard UNE EN 12390-11 [26]:

(2) $$C_{x}=C_{i}+\left(C_{s}+C_{i}\right)\left(1-\mathrm{erf}\left[\frac{x}{2\sqrt{D_{nss}t}}\right]\right)$$

**Figure 6:**
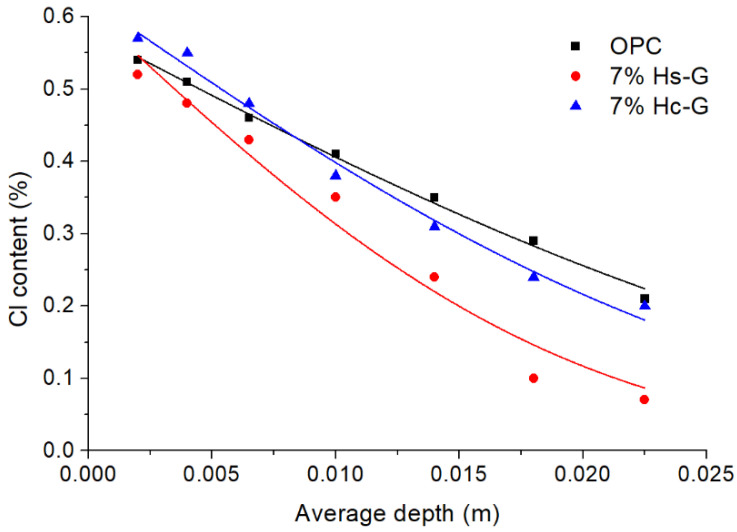
Chloride ion concentration with depth (experimental data fitted to Equation (2)).

In Equation (2), *C_x_* is chloride ion concentration at depth *x* at time *t*; *C_i_* and *C_s_*, respectively, are initial chloride content and the content on the exposed surface; and erf represents the error function. The fitted curves are plotted in Figure 6 and the parameters obtained are given in Table 6. The findings clearly revealed that the mortars with 7% replacement with a binary blend of CDW exhibited greater resistance to chloride penetration than the reference, with a 65% lower diffusion coefficient in 7% HS-G and 33% lower in 7% Hc-G. Such performance is consistent with the behavior observed in other pozzolanic additions [40,41,42,43,44,45,46], where the rate of chloride transport has customarily been associated with either enhanced chloride binding, lower matrix permeability, or both [47,48].

As noted in Section 3.1, the lower effective porosity in 7% Hs-G mortar was attributable to the greater pozzolan-induced intricacy of its pore system, an effect typically associated with a decline in the rate of ion diffusion in the material. Such lower permeability was consistent with the lower chloride ion concentration detected in mortar 7% Hs-G than in the OPC mortar at all the layers analyzed. Although mortar 7% Hc-G had lower porosity than the OPC material, the observation of higher chloride concentration values in the outermost layers (<8 mm) in the former than in the latter suggests that chemically- or physically-mediated chloride binding, rather than pore structure, played a determinant role in reducing the ion transport rate in this type of mortar. As in earlier studies, here mortars bearing calcareous CDW-based pozzolans were observed to form greater amounts of AFm-type phases, such as carboaluminates (Ca_4_Al_2_(CO_3_)·11H_2_O and Ca_4_Al_2_(CO_3_)_0.5_·12H_2_O) [20]. Such substances are known to react with chloride ions to form Friedel’s salt (Ca_4_Al_2_Cl_2_·10H_2_O), thereby immobilizing these ions in the outer layers and explaining the behavior of 7% Hc-G mortars in this regard [49,50,51]. In addition, chloride ions may also be physically adsorbed by C-S-H gels, a mechanism favored in the presence of gels with a higher Ca/Si ratio [45,52]. That is yet another indication of greater physical adsorption-governed ion binding capacity in 7% Hc-G mortars, as earlier research found their C-S-H gels to have a higher Ca/Si ratio [20].

## 4. Conclusions

The conclusions that can be drawn from the present study are set out below.

➢ When added to OPC at a replacement ratio of 7%, binary CDW mortars bearing a 2:1 (by cement weight) blend of calcareous or siliceous concrete fines and shatterproof glass performed differently relative to the reference cement depending on whether the recycled concrete was calcareous (Hc) or siliceous (Hs).
➢ The presence of CDW in the blended mortars lowered water absorption by 9.2% to 17%, depending on the nature of the recycled aggregate.
➢ The same pattern was observed for effective porosity, which was 5.2% lower than the reference in 7% Hc-G and 8.7% lower in 7% Hs-G.
➢ Although the maximum and mean penetration depths were greater in the blended cement mortars than in the reference mix, the former were Spanish structural code-compliant.
➢ The accelerated carbonation test (with 3% CO_2_) showed that adding 7% of the binary blend of CDW led to slightly higher (1–3 mm) mean carbonation penetration than in the unadditioned reference.
➢ Non-steady state resistance to chloride ion aggression was greater in the 7% blended mortars than in the OPC, although the Hs (siliceous-concrete bearing) samples performed better than their calcareous counterparts. The diffusion coefficients obtained by applying Fick’s law were 2.9 × 10^−11^ m^2^ s^−1^ for 7% Hs-G, 1.5 × 10^−11^ m^2^ s^−1^ for 7% Hc-G and 4.3 × 10^−11^ m^2^ s^−1^ for OPC.

The present findings support the premise that replacing OPC with a 2:1 binary blend of concrete fines (<5 mm) and shatterproof glass at a ratio of 7% does not compromise the durability of the resulting mortar. In fact, for most of the properties analyzed such mortars performed better than the reference material. These recycled products would consequently be usable to prepare future cement matrices with a lower clinker content and smaller carbon footprint. Such a promising future notwithstanding, further research would be recommended, primarily to test performance at higher replacement ratios than explored here.

## Figures and Tables

**Table 1:** Mineralogical composition of the materials quantified by the Rietveld method.

Phase (%)	OPC	Hc	Hs	G
Alite (C_3_S)	52	-	-	-
Belite (C_2_S)	20	-	-	-
C_3_A	9	-	-	-
C_4_AF	6	-	-	-
Calcite	4	52	24	-
Mica	-	10	4	-
Feldspars	-	11	8	-
Quartz	-	10	48	-
Am. matter	9	17	16	100
Total	100	100	100	100

**Table 2:** Laser-determined D10, D50 and D90 values for the samples studied.

µm	OPC	7% Hc-G	7% Hs-G	Hc-G	Hs-G	G
D10	1.99	1.94	1.95	1.41	1.65	2.70
D50	11.60	10.90	11.30	6.82	9.99	12.40
D90	34.20	33.90	34.60	34.60	41.60	41.40

**Table 3:** Physical and mechanical parameters of the mortars under study.

Mortar	TSA (%)	TSBA (%)	BD (g/cm^3^)	DD (g/cm^3^)	EP (%)	Compressive Strength (MPa)
OPC	7.6	8.3	2.07	2.48	17.2	69.5
7%Hc-G	6.9	7.8	2.08	2.49	16.3	66.6
7%Hs-G	6.3	7.4	2.10	2.50	15.7	66.5

**Table 4:** Depth of water penetration under pressure (mm).

Mortar	Maximum Depth (mm)	Mean Depth (mm)
OPC	19	15
7% Hc-G	28	18
7% Hs-G	28	25
DEE specification	≤50	≤30

**Table 5:** Mean carbonation depth and standard deviation (SD).

Mortar	Mean Depth (mm)	SD
OPC	8.0	1.5
7% Hc-G	11.0	1.3
7% Hs-G	9.2	2.1

**Table 6:** Chloride diffusion coefficients.

	OPC	7% Hc-G	7% Hs-G
Dnss (m^2^/s)	4.33 × 10^−11^	2.89 × 10^−11^	1.51 × 10^−11^
Error	2.26 × 10^−12^	2.30 × 10^−12^	2.29 × 10^−12^
Cs (%)	0.58	0.62	0.61
R^2^	0.995	0.988	0.967
